# Supplementary material for: A randomized feasibility trial of time-restricted eating during pregnancy in people with increased risk of gestational diabetes
Source: Sci Rep. 2024 Sep 28;14:22476. doi: 10.1038/s41598-024-72913-y (PMC11439041; doi:10.1038/s41598-024-72913-y)
Supplement: Supplementary file 1 — Supplementary Material 1 [file 41598_2024_72913_MOESM1_ESM.pdf]

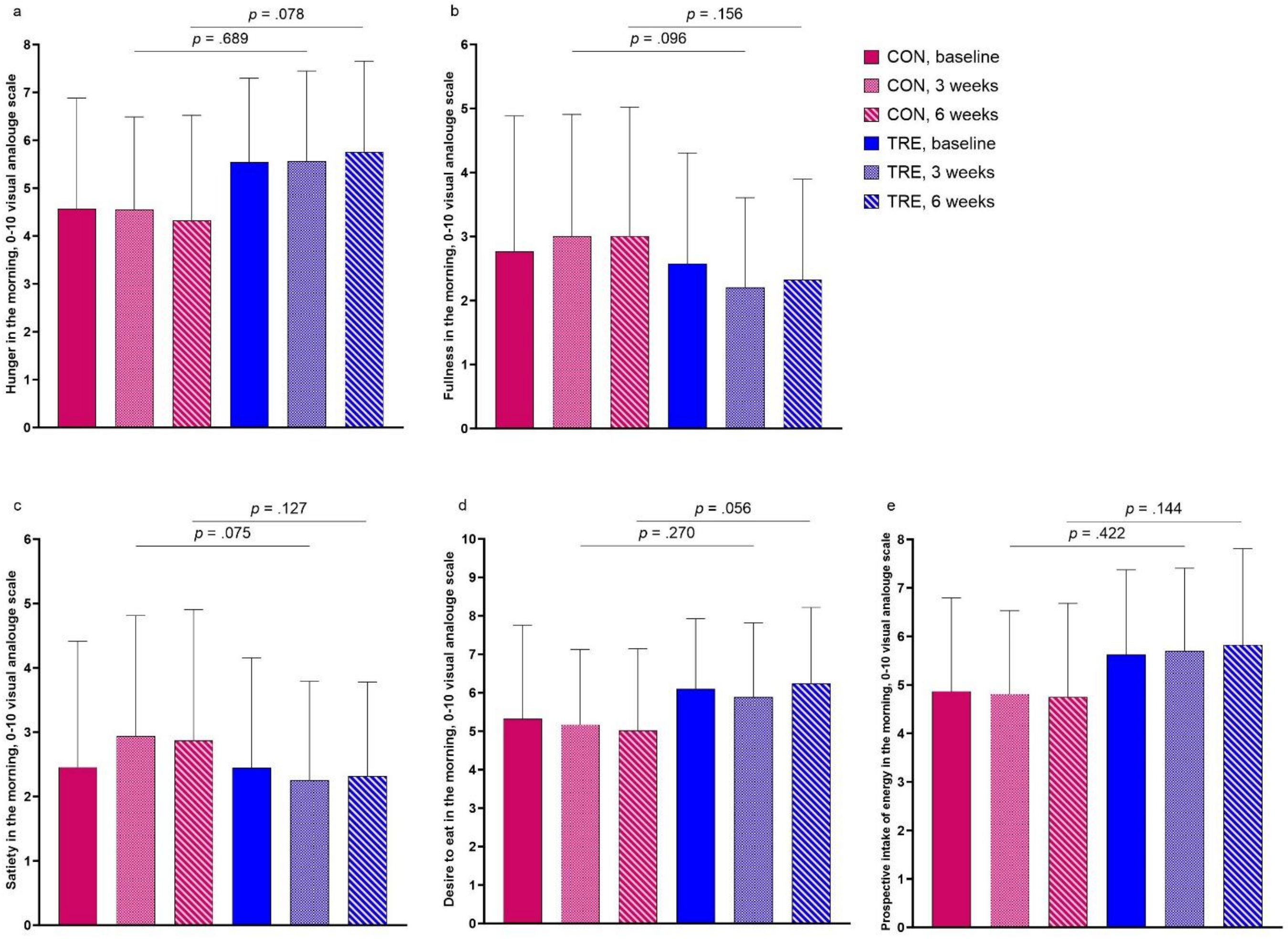

Supplementary Figure S1. Self-reported appetite in the morning. Feelings of a) hunger, b) fullness, c) satiety, d) desire to eat, and e) prospective intake of energy, as indicated on a 0-10 visual analogue scale in the morning before the first energy intake. The data are observed mean scores at baseline, in the second intervention week (3 weeks) and in the last intervention week (6 weeks). Bars show averages and error bars show standard deviations. *p* – values are for between-group comparisons using linear mixed models. CON = Control group, TRE = Time-restricted eating.

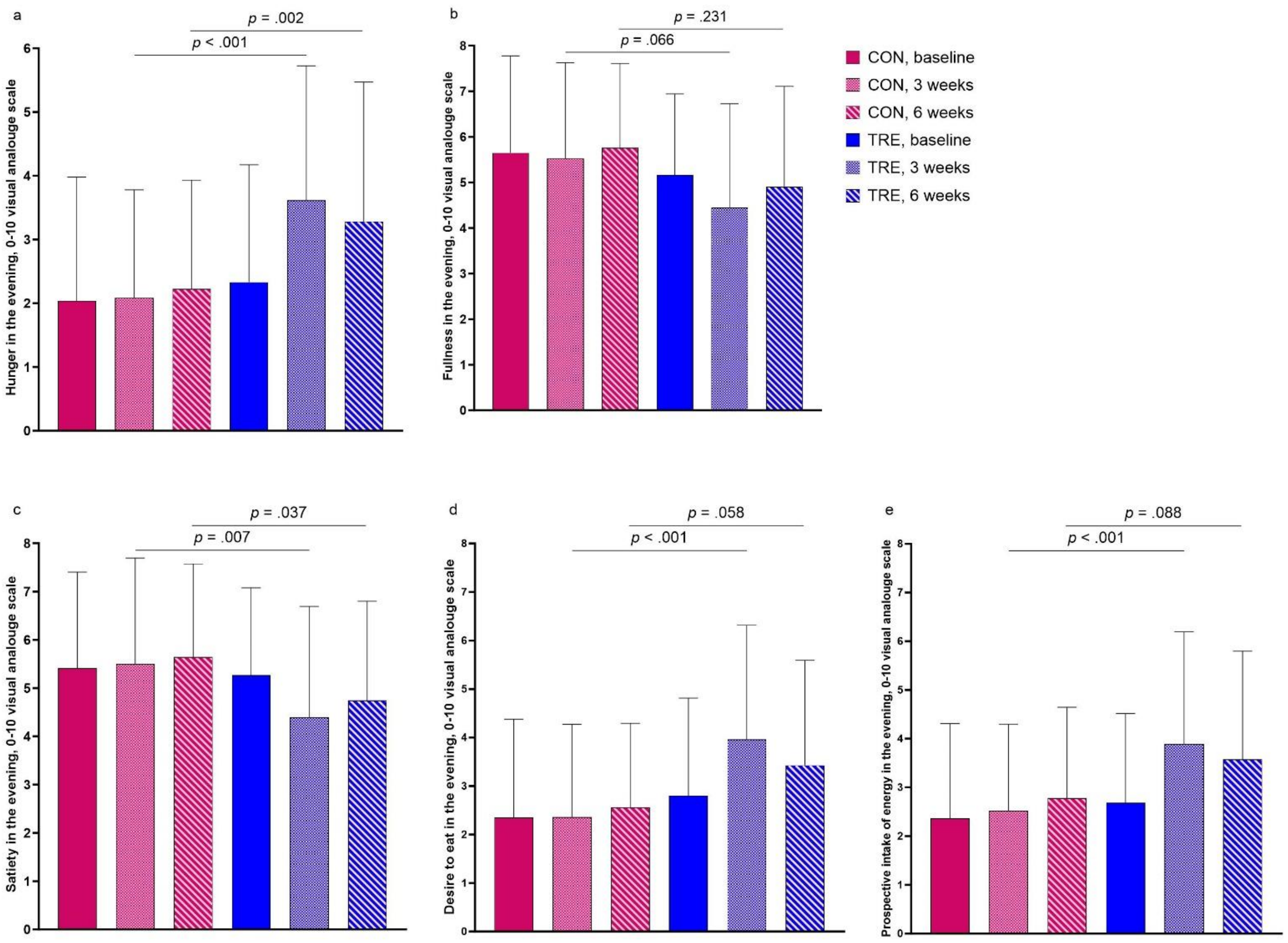

Supplementary Figure S2. Self-reported appetite in the evening. Feelings of a) hunger, b) fullness, c) satiety, d) desire to eat, and e) prospective intake of energy, as indicated on a 0-10 visual analogue scale in the evening before going to bed. The data are observed mean scores at baseline, in the second week of the intervention period (3 weeks) and in the last week of the intervention period (6 weeks). Graphs show averages and error bars show standard deviations. *p* – values are for between-group comparisons using linear mixed models. CON = Control group, TRE = Time-restricted eating.

Supplementary Table S1. Ratings of hunger and appetite on visual analogue scales in the morning and in the evening. Data are observed values with standard deviation (SD) at baseline (Week 1), the second week of the intervention (Week 3), and in the last week of the intervention (Week 6). Between-group differences are estimated using linear mixed models and reported as estimated (est.) effects with 95% confidence intervals (CI) and p-values.

		Week 1	Week 3	Week 6	Between-group difference Week 3			Between-group difference Week 6		
**Outcome**	**Group**	**Mean (SD)**	**Mean (SD)**	**Mean (SD)**	**Est. effect**	**95% CI**	**p**	**Est. effect**	**95% CI**	**p**
**Morning ratings**										
Hunger	CON	4.6 (2.3)	4.6 (1.9)	4.3 (2.2)						
	TRE	5.5 (1.6)	5.6 (1.9)	5.6 (1.9)	0.1	-0.5 to 0.7	.689	0.5	-0.1 to 1.2	.078
Fullness	CON	2.8 (2.1)	3.0 (1.9)	3.0 (2.0)						
	TRE	2.6 (1.7)	2.2 (1.4)	2.3 (1.6)	-0.4	-0.9 to 0.1	.096	-0.4	-0.9 to 0.1	.156
Satiety	CON	2.5 (2.0)	2.9 (1.9)	2.9 (2.0)						
	TRE	2.5 (1.7)	2.3 (1.5)	2.3 (1.5)	-0.4	-0.9 to 0.1	.075	-0.4	-0.9 to 0.1	.127
Desire to eat	CON	5.3 (2.4)	5.2 (2.0)	5.0 (2.1)						
	TRE	6.1 (1.8)	5.9 (1.9)	6.3 (2.0)	0.1	-0.5 to 0.7	.270	0.6	-0.02 to 1.2	.056
Prospective intake	CON	4.9 (1.9)	4.8 (1.7)	4.8 (1.9)						
	TRE	5.6 (1.8)	5.7 (1.7)	5.8 (2.0)	0.2	-0.2 to 0.8	.422	0.4	-0.1 to 0.9	.144
**Evening ratings**										
Hunger	CON	2.0 (1.9)	2.1 (1.7)	2.2 (1.7)						
	TRE	2.3 (1.8)	3.6 (2.1)	3.3 (2.2)	1.5	0.9 to 2.1	<.001	0.9	0.3 to 1.5	.002
Fullness	CON	5.7 (2.1)	5.5 (2.1)	5.8 (1.9)						
	TRE	5.2 (1.8)	4.5 (2.3)	4.9 (2.2)	-0.6	-1.3 to 0.04	.066	-0.4	-1.1 to 0.3	.231
Satiety	CON	5.4 (2.0)	5.5 (2.2)	5.6 (1.9)						
	TRE	5.3 (1.8)	4.4 (2.3)	4.8 (2.1)	-0.9	-1.6 to -0.3	.007	-0.7	-1.4 to -0.04	.037
Desire to eat	CON	2.4 (2.0)	2.4 (1.9)	2.6 (1.7)						
	TRE	2.8 (2.0)	4.0 (2.4)	3.5 (2.2)	1.5	0.8 to 2.1	<.001	0.6	-0.02 to 1.3	.058
Prospective intake	CON	2.4 (2.0)	2.5 (1.8)	2.8 (1.9)						
	TRE	2.7 (1.8)	3.9 (2.3)	3.6 (2.2)	1.1	0.5 to 1.7	<.001	0.5	-0.08 to 1.2	.088

Supplementary Table S2. Glucose area under the curve (AUC) for 24 hours (24-h), during the daytime (Day) and night-time (Night), estimated from continuous glucose monitors. Data are observed values with standard deviation (SD) at baseline, the second week of the intervention (Week 3), and in the last week of the intervention (Week 6). Between-group differences are estimated using linear mixed models and reported as estimated (est.) effects with 95% confidence intervals (CI) and p-values.

		Week 1	Weeks 2-4	Weeks 5-6	Between-group difference Week 3			Between-group difference Week 6		
**Outcome**	**Group**	**Mean (SD)**	**Mean (SD)**	**Mean (SD)**	**Est. effect**	**95% CI**	***p***	**Est. effect**	**95% CI**	***p***
24-h AUC, mmol/L	CON	4.5 (0.4)	4.5 (0.4)	4.4 (0.5)						
	TRE	4.5 (0.9)	4.6 (0.2)	4.8 (0.4)	0.01	-0.2 to 0.3	.953	0.24	-0.01 to 0.5	.059
Day AUC, mmol/L	CON	4.7 (0.4)	4.6 (0.4)	4.5 (0.5)						
	TRE	4.6 (0.9)	4.8 (0.3)	5.0 (0.4)	0.02	-0.2 to 0.3	.855	0.24	-0.02 to 0.5	.074
Night AUC, mmol/L	CON	4.0 (0.5)	4.1 (0.3)	4.0 (0.5)						
	TRE	3.9 (0.8)	4.1 (0.2)	4.1 (0.4)	-0.07	-0.3 to 0.2	.624	0.11	-0.2 to 0.4	.442

Supplementary Table S3. Self-reported daily dietary intake. Data are observed values with standard deviation (SD) at baseline (Week 1), the second week of the intervention (Week 3), and in the last week of the intervention (Week 6). Between-group differences are estimated using linear mixed models and reported as estimated (est.) effects with 95% confidence intervals (CI) and p-values.

		Week 1	Week 3	Week 6	Between-group difference Week 3			Between-group difference Week 6		
**Outcome**	**Group**	**Mean (SD)**	**Mean (SD)**	**Mean (SD)**	**Est. effect**	**95% CI**	***p***	**Est. effect**	**95% CI**	***p***
Total energy intake, kJ	CON	9285 (2492)	9281 (3202)	8855 (2415)						
	TRE	9012 (1293)	8884 (2280)	8920 (2223)	-15	-970 to 940	.975	-184	-1139 to 772	.701
Fat, g	CON	91 (30)	89 (40)	80 (29)						
	TRE	85 (19)	86 (32)	90 (32)	5	-10 to 21	.496	9	-7 to 24	.264
Saturated fat, g	CON	37 (16)	37 (19)	31 (13)						
	TRE	34 (9)	33 (15)	34 (15)	-4	-10 to 3	.256	2	-4 to 8	.548
Cis-mono-unsaturated fat, g	CON	33 (12)	32 (17)	29 (13)						
	TRE	30 (6)	32 (16)	34 (14)	5	-4 to 13	.276	4	-4 to 13	.341
Cis-poly-unsaturated fat, g	CON	12 (4)	11 (5)	12 (7)						
	TRE	13 (4)	13 (6)	14 (6)	3	-0.3 to 5	.081	1	-2 to 4	.483
Carbohydrates, g	CON	245 (74)	255 (100)	254 (79)						
	TRE	241 (34)	240 (77)	227 (71)	-14	-46 to 18	.388	-33	-65 to -2	.040
Sugar, g	CON	40 (26)	66 (58)	42 (27)						
	TRE	53 (50)	37 (35)	31 (28)	-34	-58 to -11	.005	-16	-40 to 9	.198
Fiber, g	CON	24 (6)	23 (10)	24 (9)						
	TRE	25 (5)	22 (7)	24 (8)	-2	-5 to 1	.187	-2	-5 to 1	.277
Protein, g	CON	93 (17)	92 (30)	85 (28)						
	TRE	92 (11)	86 (28)	91 (26)	-3	-12 to 6	.513	1	-8 to 11	.792

kJ = kilojoules, TEI = Total energy intake.

Supplementary Table S4. Daily physical activity, estimated from activity armbands. Data are observed values with standard deviation (SD) at baseline (Week 1), the second week of the intervention (Week 3), and in the last week of the intervention (Week 6). Between-group differences are estimated using linear mixed models and reported as estimated (est.) effects with 95% confidence intervals (CI) and p-values.

		Week 1	Week 3	Week 6	Between-group difference Week 3			Between-group difference Week 6		
**Outcome**	**Group**	**Mean (SD)**	**Mean (SD)**	**Mean (SD)**	**Est. effect**	**95% CI**	***p***	**Est. effect**	**95% CI**	***p***
Physical activity level, METs	CON	1.2 (0.2)	1.3 (0.2)	1.3 (0.1)						
	TRE	1.3 (0.2)	1.3 (0.3)	1.4 (0.4)	-0.01	-0.2 to 0.2	.934	0.1	-0.1 to 0.3	.188
Daily energy expenditure, kJ	CON	8888 (1513)	8967 (1490)	8498 (1421)						
	TRE	9176 (962)	9430 (911)	10245 (602)	63	-765 to 890	.879	794	-68 to 1655	.070
Sedentary time, min	CON	1068 (184)	943 (183)	960 (243)						
	TRE	920 (209)	927 (231)	995 (289)	16	-106 to 138	.789	-31	-157 to 96	.627
Light intensity activity, min	CON	142 (62)	183 (141)	165 (93)						
	TRE	166 (111)	180 (114)	197 (86)	1	-63 to 64	.981	12	-54 to 77	.723
Moderate intensity activity, min	CON	50 (45)	52 (48)	42 (30)						
	TRE	49 (35)	57 (51)	75 (66)	7	-24 to 37	.660	26	-5 to 58	.097
Vigorous intensity activity, min	CON	4 (9)	3 (6)	3 (5)						
	TRE	2 (2)	4 (7)	6 (8)	1	-4 to 7	.577	4	-2 to 9	.149

METs = Metabolic equivalent of task, PA = physical activity
